# Identification of an NF-κB p50/p65-responsive site in the human *MIR155HG* promoter

**DOI:** 10.1186/1471-2199-14-24

**Published:** 2013-09-23

**Authors:** Ryan C Thompson, Iosif Vardinogiannis, Thomas D Gilmore

**Affiliations:** 1 Department of Biology, Boston University, Boston, MA 02215, USA

**Keywords:** miR-155, MIR155HG, BIC, NF-kappaB, Promoter, Transcriptional activation

## Abstract

**Background:**

MicroRNA-155 (miR-155) is the diced product of the *MIR155HG* gene. miR-155 regulates the expression of many immune-specific transcripts, is overexpressed in many human lymphomas, and has oncogenic activity in mouse transgenic models. *MIR155HG* has been proposed to be a target gene for transcription factor NF-κB largely due to the positive correlation between high nuclear NF-κB activity and increased miR-155 expression following treatment with NF-κB inducers or in subsets of hematopoietic cancers. Nevertheless, direct regulation of the human *MIR155HG* promoter by NF-κB has not been convincingly demonstrated previously.

**Results:**

This report shows that induction of NF-κB activity rapidly leads to increased levels of both primary *MIR155HG* mRNA and mature miR-155 transcripts. We have mapped an NF-κB-responsive element to a position approximately 178 nt upstream of the *MIR155HG* transcription start site. The -178 site is specifically bound by the NF-κB p50/p65 heterodimer and is required for p65-induced reporter gene activation. Moreover, the levels of miR-155 in nine human B-lymphoma cell lines generally correlate with increased nuclear NF-κB proteins.

**Conclusion:**

Overall, the identification of an NF-κB-responsive site in the *MIR155HG* proximal promoter suggests that *MIR155HG* is a direct NF-κB target gene *in vivo*. Understanding NF-κB-mediated regulation of miR-155 could lead to improved immune cell-related diagnostic tools and targeted therapies.

## Background

miR-155 is one of the most extensively studied miRs due to its involvement in immune cell development and function and in various diseases [1-4]. Increased miR-155 expression is typically seen following the treatment of cells with proinflammatory signaling molecules like lipopolysaccharide (LPS), interleukins, interferons, and tumor necrosis factor [5-9]. Mice with a knockout of miR-155 have dysfunctional lymphocytes and dendritic cells [10,11]. Increased miR-155 expression is also observed in many inflammatory diseases such as multiple sclerosis and rheumatoid arthritis [9], and in many B- and T-lymphomas and leukemias [2,12-15]. Transgenic mice with tissue-specific expression of miR-155 can develop hematopoietic malignancies [16,17].

The mature form of miR-155 is encoded by the *MIR155HG* gene (formerly called *BIC* [*B-cell Integration Cluster*]) [18,19]. Although *MIR155HG* encodes two miRs (miR-155-5p and miR-155-3p), miR155-5p is the more commonly studied product, and herein will be referred to as miR-155. miR-155 is excised from an exon of its pre-miRNA precursor by Dicer and then loaded into the RISC complex [2,19]. This miR-155/RISC complex can then bind to mRNA transcripts with miR-155 target sequences in their 3`-UTRs. miR-155-regulated transcripts include ones encoding PU.1, AID, IKKϵ, C/EBPβ, SOCS1, MITF, and FADD [1,7,8,20], and miR-155 has been shown to decrease translation of these target mRNAs.

To date, the majority of evidence that transcription factor NF-κB activates miR-155 expression is circumstantial, extrapolated from the positive correlation between high nuclear NF-κB activity and increased miR-155 expression in both normal lymphoid cells and various cancers, including many hematopoietic malignancies [7,12-15]. Several studies have set out to identify the *cis* regulatory elements in the *MIR155HG* promoter, producing mixed and sometimes conflicting results. One study claims that EBV-induced *MIR155HG* expression is primarily regulated by an AP-1 site found 40 nt upstream of the transcription start site (TSS) [21], while another concludes that EBV induces *MIR155HG* through two NF-κB sites over 1100 nt upstream of the TSS [22]. A third study suggests that NF-κB up-regulates AP-1 components, which then bind to the AP-1 site (at -40) to up-regulate miR-155 expression [23]. Thus, no study has convincingly established *MIR155HG* as a direct target of NF-κB.

In this report, we show that NF-κB p50/65 can directly bind to and activate the human *MIR155HG* proximal promoter through a site approximately 178 nt upstream of the *MIR155HG* TSS. Furthermore, we demonstrate that *MIR155HG*/miR-155 expression and p50/p65 binding to this site are rapidly induced after treatment of the B-lymphoma cell line BJAB with the NF-κB inducer LPS. Lastly, in a panel of B-lymphoma cell lines, nuclear NF-κB protein levels generally correlate with increased miR-155 expression. The results of this study show that miR-155 expression can be directly controlled by NF-κB through a site in the *MIR155HG* proximal promoter.

## Methods

### Plasmids

The firefly luciferase reporter plasmid containing 1494 nt region upstream of the *MIR155HG* TSS pWT-MIR155HG), the -1150 κB-site mutant plasmid (-1150mut-MIR155HG), and the AP-1mut-MIR155HG were gifts of Eric Flemington and have been described previously [21]. The -441 (-441mut-MIR155HG) and -178 (-178mut-MIR155HG) mutants were created by insertion of an *Xho*I site in the middle of the given κB site, as in -1150mut-MIR155HG (see Additional file 1: Table S1). Reporter plasmids with truncations at -531 and -91 were created by amplifying the relevant regions of the *MIT155HG* promoter (see Additional file 1: Table S1) and subcloning them into *NheI*/*HindIII*-digested pWT-MIR155HG. pcDNA-FLAG-p65 and pcDNA-FLAG-p50 were created by subcloning PCR-amplified sequences of each into the *XhoI* site of pcDNA-FLAG. pcDNA-REL has been described previously [24].

### Cell culture

The cell lines used in this study were A293 human embryonic kidney cells, COS-1 monkey kidney cells, 3T3 and 3T3*nfkb1*^-/-^*nfkb2*^-/-^*relb*^-/-^*crel*^-/-^ (gift of Alexander Hoffmann) mouse fibroblast cells, and the following human B-lymphoma cell lines: GCB diffuse large B-cell lymphoma BJAB, SUDHL4, and Pfeiffer [25-27]; follicular lymphoma BL41 [28]; Burkitt’s lymphoma Daudi and Ramos [29,30]; Hodgkin lymphoma KMH2 and L428 [31]; LMP1^+^ B-lymphoblastoid IB4 [28]. All cell lines were cultured in Dulbecco’s modified Eagle’s medium (DMEM; Life Technologies) or RPMI medium 1640 (Life Technologies) supplemented with 10-20% heat-inactivated fetal bovine serum (FBS) (Biologos) as previously described [7,32].

For transfections, A293 and COS-1 cells were seeded in 24-well, 35-mm, or 60-mm tissue culture dishes such that they were approximately 60% confluent on the following day when transfections were performed using polyethylenimine (PEI) (Polysciences) [7]. On the day of transfection, DNA:PEI mixtures of 1:3 (A293 cells) and 1:12 (COS-1 cells) were incubated for 15 min at room temperature (RT), then added to cells, and incubated overnight.

For transfections of 3T3 and 3T3*nfkb1*^-/-^*nfkb2*^-/-^*relb*^-/-^*crel*^-/-^ cells, the cells were seeded in a 24-well dish at a density of 0.75 × 10^5^ on the day of transfection. Prior to plating cells, transfection mixtures containing a plasmid DNA:Enhancer ratio of 1:5.5 were incubated for 5 min at RT and these mixtures were incubated for 30 min at RT containing a DNA:Effectene ratio of 1:7.9 (Qiagen). After the 30 min incubation, cells were plated, the transfection mixtures were immediately added to each well, and cells were incubated overnight.

For both PEI and Effectene transfections, the day after transfection the transfection media was replaced with fresh DMEM containing 10% FBS. Cells were harvested and lysed 24 h later for the appropriate assay.

### Western blotting

Western blotting was performed as described previously [7,33]. Whole cell extracts were prepared in AT buffer (20 mM HEPES, pH 7.9, 1 mM EDTA, 1 mM EGTA, 20 mM Na_4_P_2_O_7_, 1 mM DTT, 1% v/v Triton X-100, 20% w/v glycerol, 1 mM Na_3_VO_4_, 1 μg/ml PMSF, 1 μg/ml leupeptin, 1 μg/ml pepstatin) or nuclear extracts were prepared as described below for EMSAs. Samples were boiled for 10 min in SDS sample buffer (62.5 mM Tris–HCl, pH 6.8, 3.2% w/v SDS, 10% w/v glycerol, 5% v/v β-mercaptoethanol, 0.1% w/v bromophenol blue). Samples containing equal amounts of protein were separated on SDS-polyacrylamide gels and transferred to nitrocellulose membranes (Micron Separation Inc.). The following primary antisera were used: p65 (1:2000, kind gift of Nancy Rice, #1226); PARP (1:500, Santa Cruz Biotechnology, #1750); β-tubulin (1:500, Santa Cruz Biotechnology, #9104); FLAG (1:1000, Cell Signaling Technology, #2368); REL (1:1000, kind gift of Nancy Rice, #265); and p50 (1:500, Santa Cruz Biotechnology, #114). Nitrocellulose filters were incubated with primary antiserum for 1 or 18 h at room temperature or 4°C, respectively. The appropriate horseradish peroxidase-labeled secondary antiserum was added and immunoreactive proteins were detected with the SuperSignal Dura West Extended Duration Substrate chemiluminescence detection system (Thermo Fisher Scientific).

### Luciferase reporter assays

Luciferase reporter assays were performed using the Dual-Luciferase Assay System (Promega) as previously described [34]. COS-1, 3T3, and 3T3*nfkb1*^-/-^*nfkb2*^-/-^*relb*^-/-^*crel*^-/-^ cells were plated in 24-well dishes and co-transfected with 0.2 μg of the appropriate pGL3-reporter plasmid, 15–30 ng of the transfection normalization plasmid RSV-Renilla-luciferase (gift of Anthony Faber), and 0.4 μg of pcDNA-REL or FLAG-tagged versions of pcDNA-p65, -p50, or vector alone. Two days later, cells were lysed, firefly and renilla luciferase activities were determined, and values were normalized to the relevant vector control (1.0).

### Real-time quantitative PCR (qPCR)

Total RNA was isolated using TRIzol (Invitrogen) and real-time quantitative PCR (qPCR) was performed as described previously [7,32] with the primer sets listed in Additional file 1: Table S1. qPCR for mature miR-155 and 5S ribosomal RNA was performed by first reverse transcribing the RNA with specific stem-loop primers that contain an annealing site for the universal reverse primer (Additional file 1: Table S1) using the TaqMan miRNA Reverse Transcriptase kit (Applied Biosystems) as described previously [7,35]. Mature miR-155 and 5S sequences were then amplified using a forward-specific primer and a universal reverse primer (Additional file 1: Table S1). The qPCR reaction was performed using the 7900HT fast real-time PCR system (Life Technologies) with the Power SYBR Green PCR Master Mix (Life Technologies). Quantification of miR-155 by qPCR was performed three times with triplicate samples and values were normalized to the 5S values. The final values represent relative expression (miR-155/5S) as compared to untreated control samples (for LPS treatment of BJAB cells) and Ramos cells (for the panel of lymphoma cell lines). For quantification of the unspliced form of *MIR155HG*, total RNA was isolated as above and whole cell cDNA preps were made using M-MLV reverse transcriptase (Promega). Unspliced *MIR155HG* and *GAPDH* cDNAs were amplified using specific forward and reverse primers (Additional file 1: Table S1), and qPCR was performed as above.

### Electrophoretic mobility shift assays (EMSAs)

EMSAs were performed using 10–20 μg of nuclear extract prepared from A293 cells transfected with FLAG-tagged versions of pcDNA, pcDNA-p65, or pcDNA-p50, or from BJAB cells treated with LPS, generally as described previously [32,33]. Nuclear extracts were incubated with 2 μg poly(dI-dC), and ^32^P-labeled *MIR155HG*-178 or *MHC1* NF-κB site probes (Additional file 1: Table S1) in binding buffer (25 mM Tris–HCl, pH 7.4, 100 mM KCl, 0.5 mM EDTA, 6.25 mM MgCl_2_, 0.5 mM DTT, 10% w/v glycerol) in a final reaction volume of 50 μl. DNA-binding reactions were carried out for 30 min at room temperature. Supershifts were performed using two p65 antibodies (#372x, Santa Cruz Biotechnology and #1226, kind gift of Nancy Rice) or normal rabbit serum, which were incubated with the protein-DNA complexes for an additional 1 h on ice. Samples were resolved on 5% non-denaturing polyacrylamide gels. Gels were dried and protein-DNA complexes were detected by autoradiography.

### Prediction of transcription factor binding sites in the *MIR155HG* promoter

Prediction of NF-κB transcription factor binding sites in the human *MIR155HG* promoter was performed using the online prediction models PROMO [36] and the Transcriptional Regulatory Element Database (TRED) [37]. The two sites with high scores in both programs---the previously described -1150 site [21] and the -178 site---as well as the -441 site that is conserved in the mouse *MIR155HG* upstream region were selected for future experiments.

### Statistical analysis

Standard error was calculated for all data. P-values were calculated using an ANOVA test.

## Results

### LPS treatment of BJAB cells induces p65 nuclear translocation, increased *MIR155HG*/miR-155 transcript expression, and increased DNA binding to a site in the *MIR155HG* proximal promoter

**Figure 1:**
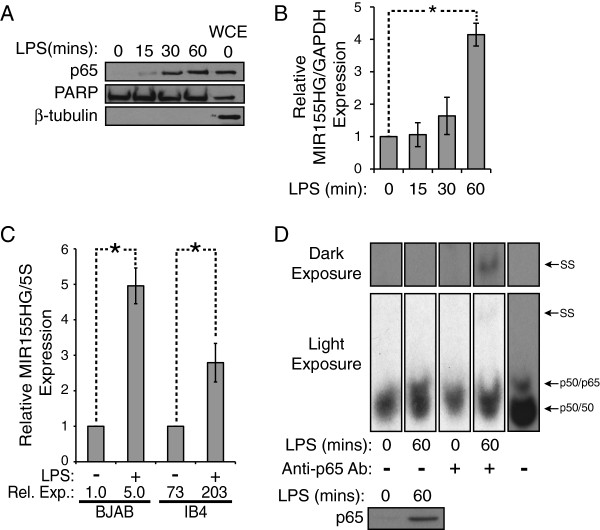
**LPS treatment leads to increased p65-directed miR-155 expression. (A)** BJAB cells were treated with 5 μg/ml LPS for 0, 15, 30, and 60 min and nuclear extracts were made. Western blots were performed on p65, PARP (nuclear control), and β-tubulin (cytosolic control). Whole cell extracts (WCE) of BJAB cells were used as a control. **(B)** BJAB cells were treated with 5 μg/ml LPS as in **(A)** and cDNA preps were prepared. The expression levels of the unspliced *MIR155HG* transcript were assessed by qPCR and normalized to GAPDH. Values were normalized to untreated cells (1.0), +/- SE and * indicates p < 0.003. **(C)** BJAB and IB4 cells were treated with 5 μg/ml LPS for 24 h and cDNA preps for small RNAs were prepared. The mature miR-155 expression levels were then assessed by qPCR and normalized to 5S rRNA. Values were normalized to untreated samples for each cell type and the relative expression (Rel. Exp.) is relative to untreated BJAB cells. The * indicates p < 0.003. **(D)** BJAB cells were treated with 5 μg/ml LPS for 0 or 60 min and nuclear extracts were made. An EMSA was performed using a ^32^P-labeled probe containing the predicted -178 NF-κB site in the *MIR155HG* promoter (described in Figures 2 and 3). Where indicated, an antibody specific for p65 was added. A light exposure shows p50/p65 heterodimer binding just above the p50 homodimer band, and the darker exposure shows the supershift with p65 antibody. The last lane is a size control made from a whole cell extract of A293 cells transfected with p65 and p50 plasmids (refer to Figure 3). Nuclear extracts were also subjected to anti-p65 Western blotting (bottom panel).

miR-155 has often been shown to be up-regulated by inducers of NF-κB activity [5-8]. To determine whether *MIR155HG* is an early response gene in LPS-induced NF-κB signaling, we first measured the kinetics of p65 nuclear translocation following LPS treatment. BJAB cells, which contain low levels of nuclear p65, were treated with 5 μg/ml LPS for increasing times up to 60 min and the levels of nuclear p65 were measured by Western blotting (Figure 1A). Within 15 min of LPS treatment, p65 started to increase in the nucleus, and by 60 min, there was a substantial amount of p65 in the nucleus. As controls, we show that PARP is present in nuclear extracts, β-tubulin (a cytosolic protein) is absent from nuclear extracts, and that p65, PARP and β-tubulin are present in BJAB whole cell extracts.

To determine whether nuclear translocation of p65 coincided with increased expression of *MIR155HG*/miR-155, BJAB cells were treated with LPS for times up to 60 min as in Figure 1A, and qPCR was performed for the unspliced *MIR155HG* transcript and for the mature form of miR-155. To ensure that LPS was inducing increased levels of newly transcribed *MIR155HG* RNA, we measured the unspliced *MIR155HG* transcript using one primer located in exon 2 and one in intron 2 of the *MIR155HG* gene. After 60 min of LPS treatment, unspliced *MIR155HG* was increased by approximately four-fold as compared to untreated cells (Figure 1B). Additionally, in both BJAB and IB4 cells (B-lymphoblast cell line with high NF-κB and miR-155 expression relative to BJAB cells [7]), treatment with LPS for 24 h resulted in 5-fold and 2.8-fold increases in mature miR-155 levels, respectively (Figure 1C). Of note, IB4 cells have ~75 times more miR-155 in untreated cells as compared to untreated BJAB cells, showing that induction of miR-155 expression is not strictly dependent on the resting levels of miR-155.

To assess the DNA-binding ability of LPS-induced nuclear p65, an EMSA was performed to measure the amount of NF-κB heterodimer p50/p65 that bound to a specific site in the *MIR155HG* promoter (using the -178 probe described below). As shown in Figure 1D, nuclear extracts from untreated control BJAB cells contain high levels of the NF-κB p50/p50 homodimer that can bind to the -178 probe. Following treatment of BJAB cells with LPS for 60 min, a new DNA-protein complex appeared that migrated slightly slower than the p50/p50 homodimer band. This new, higher band co-migrated with a DNA-protein complex from extracts of A293 cells transfected with pcDNA-FLAG versions of both p50 and p65 (Figure 1D). Additionally, this new band was supershifted with p65 antibody, but not with pre-immune serum (Figure 1D). Moreover, the appearance of the p50/p65 DNA-binding activity coincides with the increased nuclear localization of p65 (Figure 1D, bottom panel). Taken together, these results suggest that LPS induces the nuclear translocation of an NF-κB p50/p65 heterodimer that can bind to a site in the *MIR155HG* promoter.

### The *MIR155HG* promoter is selectively activated by p65

**Figure 2:**
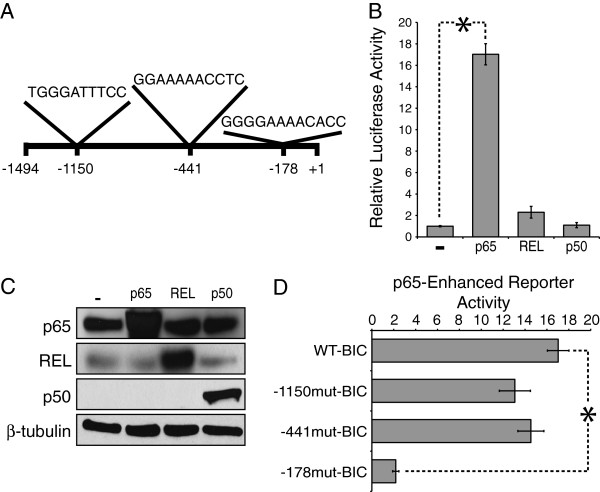
**NF-κB p65 up-regulates expression from the *MIR155HG *promoter through an NF-κB binding site located upstream of the transcription start site. (A)** At least three NF-κB binding sites are predicted in sequences within 1494 nt upstream of the *MIR155HG* transcription start site (+1): the -1150 site was previously reported [21] and the sites at -441 and -178 were predicted using the online prediction programs Promo and TRED. **(B)** Reporter assays were performed in COS-1 cells transfected with the pGL3-based wild-type *MIR155HG* promoter luciferase reporter plasmid (WT-MIR155HG) and either pcDNA alone (-), pcDNA-p65, pcDNA-REL, or pcDNA-p50. Relative luciferase values for each transfection were normalized for RSV-renilla expression and then normalized to the value for pcDNA alone (1.0), +/- SE, and the * indicates p < 0.2E-8. **(C)** The pcDNA versions of p65, REL, and p50 (used in **B**) were expressed individually in COS-1 cells and whole cell extracts were prepared. Western blotting for p65, REL, p50, and β-tubulin (normalizing control) confirmed expression of all three proteins. **(D)** Site-directed mutagenesis was performed to create mutants of each predicted NF-κB binding site, creating -1150mut-MIR155HG, -441mut-MIR155HG, and -178mut-MIR155HG reporter plasmids. Transfections and luciferase assays were performed as in **(B)**. The * indicates p < 0.002.

The 1494 nt region upstream of the *MIR155HG* TSS contains three predicted NF-κB binding sites (see Methods for site prediction methods): one previously reported at -1150 [21] and two sites at -441 and -178 (Figure 2A). To assess the ability of NF-κB to activate transcription from the *MIR155HG* promoter*,* COS-1 cells were co-transfected with the luciferase reporter plasmid WT-MIR155HG (containing the -1494 nt region) and expression plasmids for NF-κB proteins p65, REL (human c-Rel), or p50, or with an empty vector control. In these assays, p65 activated the *MIR155HG* promoter approximately 17-fold as compared to the empty vector control. REL and p50 had little to no effect on WT-MIR155HG reporter gene expression (Figure 2B). In a separate experiment, COS-1 cells were transfected with the same pcDNA-based plasmids, and Western blotting of whole cell extracts confirmed enhanced expression of p65, REL, and p50 (Figure 2C).

### An NF-κB p65-responsive site in the *MIR155HG* promoter is located at -178 bp

To identify the p65-responsive region in the *MIR155HG* promoter, 5’ truncation mutants of the upstream sequences in the WT-MIR155HG reporter plasmid were created at -530 and -91 nt upstream of the TSS (Additional file 2: Figure S1). The -530 truncation removes the -1150 κB site and leaves the -441 and -178 sites intact, while the -91 truncation removes all three predicted NF-κB binding sites. Reporter gene assays showed that the -530 truncation retained the ability to be activated by p65, whereas the -91 truncation showed a complete loss of p65-enhanced reporter activity (Additional file 2: Figure S1). These results indicate that the p65-responsive region lies between nt 530 and 91 upstream of the *MIR155HG* transcription start site.

To identify the p65-responsive site(s) located between -530 and -91, we assessed the ability of p65 to increase transcription of *MIR155HG* luciferase reporter plasmids with mutations in predicted NF-κB sites at -441 (-441mut-MIR155HG) or -178 (-178mut-MIR155HG). As a control, we also investigated the effect of p65 on a reporter with a mutation at -1150 (-1150mut-MIR155HG [21]). p65 enhanced transcription from the -1150 and -441 mutant site reporters to approximately the same extent as seen with the WT-MIR155HG plasmid (Figure 2D). In contrast, the -178 mutant site reporter was not activated by p65 (Figure 2D).

Because NF-κB has been reported to up-regulate components of the AP-1 transcription factor, which could then have effects on *MIR155HG* expression, we determined whether p65 could increase transcription of a *MIR155HG* reporter containing a mutation of the AP-1 site (AP-1mut-MIR155HG) [21]. p65 enhanced AP-1mut-MIR155HG reporter gene expression to an even greater extent than WT-MIR155HG reporter (Additional file 3: Figure S2), showing that the AP-1 site is not required for p65-based activation of the *MIR155HG* promoter.

### The p50/p65 heterodimer binds to the -178 site in the *MIR155HG* promoter to activate transcription

**Figure 3:**
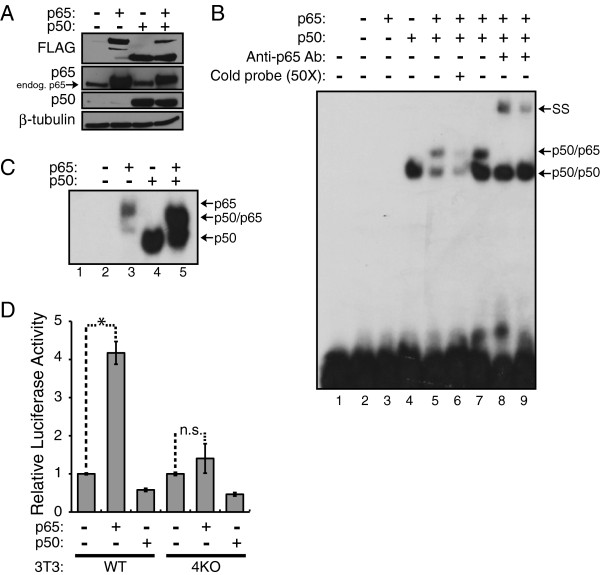
**The NF-κB p50/p65 heterodimer binds to the -178 site from the *MIR155HG *promoter to activate transcription. (A)** FLAG-tagged versions of p65 and p50 were expressed individually or together in A293 cells and whole cell extracts were prepared. Western blotting for FLAG, p65, p50, and β-tubulin (normalizing control) confirmed expression of p65 and p50. **(B)** An EMSA was performed using the extracts from **(A)** with a probe containing -178 site from the *MIR155HG* promoter (see Figure 2). Lanes are as follows: probe alone (lane 1), empty vector (lane 2), p65 alone (lane 3), p50 alone (lane 4), p50/p65 (lane 5), p50/p65 with 50X cold probe competition (lane 6), p50/p65 with pre-immune serum (lane 7), p50/p65 with SC-372x p65 antibody (lane 8), and p50/p65 with NR-1226 p65 antibody (lane 9). The supershifted p50/p65 complex is indicated by ss and an arrow. **(C)** An EMSA was performed using extracts from **(A)** with a consensus NF-κB site from the human *MHC1* enhancer (as a κB-site control [32,33,38,50]) and complexes were detected by autoradiography. The appearance of single bands in the p65 and p50 alone lanes confirms that both proteins can bind DNA. Upon co-expression of p65 and p50, a band between the p65 and p50 bands is present, representing the p50/p65 heterodimer. **(D)** Reporter assays were performed in mouse 3T3 and 3T3*nfkb1*^-/-^*nfkb2*^-/-^*relb*^-/-^*crel*^-/-^ (4KO) cells transfected with the WT-MIR155HG reported plasmid and pcDNA expression plasmids for either vector control (-), FLAG-p65, or FLAG-p50. Values for each transfection were normalized to RSV-renilla and then normalized to pcDNA alone (1.0), +/- SE. The * indicates p < 0.03, and n.s. indicates that the difference is not statistically significant.

To further investigate the ability of p65 to bind the -178 site in the *MIR155HG* promoter, an EMSA was performed using a ^32^P-labeled probe containing the -178 site and over-expressed forms of p65 and p50. For this experiment, FLAG-tagged versions of p50 and p65 were expressed individually or together in A293 cells. Western blotting with FLAG, p65, and p50 antibodies was first done on whole cell extracts to confirm over-expression of each protein after transfection (Figure 3A). The extracts from A293 cells expressing p65, p50, and p50/p65 were then used in an EMSA with the -178 site probe (Figure 3B). p65 alone did not detectably bind to the -178 site (Figure 3B, lane 3), whereas p50 alone showed strong binding to the -178 site (Figure 3B, lane 4). Using extracts from cells co-transfected with p50 and p65, a band larger than the p50 homodimer band appeared, indicating that the p50/p65 heterodimer can bind to this site (Figure 3B, lane 5). This band was competed with 50X cold probe (Figure 3B, lane 6). In addition, two anti-p65 antibodies supershifted the band that was slightly larger than the p50 alone band (Figure 3B, lanes 8 and 9). The p65 antibodies did not shift the p50 homodimer band (lanes 8 and 9), and no supershift was seen with pre-immune serum (Figure 3B, lane 7).

Because p65 alone could not bind to the -178 site probe, we wanted to make sure that the A293 cell-expressed p65 protein was able to bind DNA. Therefore, an EMSA was performed with a consensus NF-κB site from the *MHC1* enhancer, which we have previously shown can be bound by p65 and p50 [38,39]. As shown in Figure 3C, p65 and p50 (lanes 3 and 4, respectively) can individually bind the *MHC1* κB-site probe. When the p65 and p50 plasmids were co-transfected, a strong EMSA band appeared that was slightly smaller than p65 alone and slightly larger than p50 alone (Figure 3C, lane 5), which is consistent with the migration of a p50/p65 heterodimer complex.

Because p65 alone did not bind to the -178 site (Figure 3B), but transfection of p65 into COS-1 cells requires this site for induction of *MIR155HG* promoter expression (Figure 2D), we hypothesized that transfected p65 was forming heterodimers with endogenous p50 (or another NF-κB family member) to drive expression of the *MIR155HG* promoter in the reporter plasmid. Therefore, we predicted that activation of the *MIR155HG* reporter by p65 would be reduced in cells lacking other NF-κB family members. To test this prediction, we compared the ability of p65 to activate the *MIR155HG* reporter in wild-type mouse 3T3 cells and 3T3 cells lacking all NF-κB family members except p65 (3T3*nfkb1*^-/-^*nfkb2*^-/-^*relb*^-/-^*crel*^-/-^ cells). Both cell types were co-transfected with the WT-MIR155HG reporter and expression plasmids for FLAG-p65, FLAG-p50, or the empty vector control. In wild-type 3T3 cells, p65 activated the *MIR155HG* promoter approximately 4.5-fold as compared to the empty vector control, whereas in 3T3*nfkb1*^-/-^*nfkb2*^-/-^*relb*^-/-^*crel*^-/-^ cells, p65 only minimally activated luciferase activity (approximately 1.5-fold; Figure 3D). Transfection of p50 alone did not activate the *MIR155HG* reporter in either cell type. These results show that the *MIR155HG* promoter can be activated by p65 in a cell type other than COS-1 cells, and that the ability of p65 to fully activate the *MIR155HG* promoter requires other endogenous NF-κB family members.

### Activation of the NF-κB signaling pathway generally correlates with miR-155 expression in a panel of human B-lymphoma cell lines

**Figure 4:**
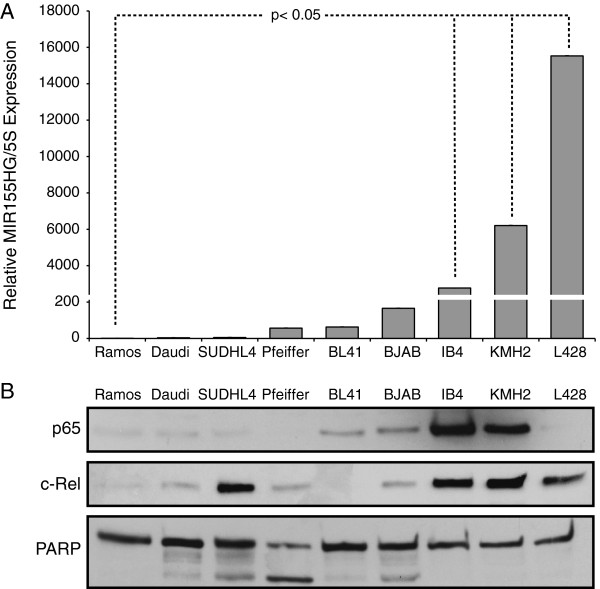
**Nuclear NF-κB proteins and expression of miR-155 in human B-lymphoma cell lines.** A panel of nine B-lymphoma cell lines was analyzed for **(A)** mature miR-155 expression by qPCR and **(B)** nuclear p65, REL, and PARP (as a normalizing control) by Western blotting. Included are cell lines known to have low NF-κB activity (Ramos, Daudi, SUDHL4, Pfeiffer, BL41, and BJAB) and cell lines known to have high NF-κB activity (IB4, KMH2, and L428) (see text and Methods). miR-155 expression values are relative to Ramos cells (1.0) and error bars indicate +/- SE.

Given that increased NF-κB activity correlates with increased miR-155 expression and that the -178 κB site can be bound by the p50/p65 heterodimer, we hypothesized that B-lymphoma cell lines with high miR-155 would also have high nuclear p65 levels in their resting state whereas B-lymphoma cell lines expressing low levels of miR-155 would have low levels of nuclear p65. Nine B-lymphoma cell lines with known NF-κB activity levels were chosen (low NF-κB: Ramos, Daudi, SUDHL4, Pfeiffer, BL41, and BJAB; high NF-κB: IB4, KMH2, and L428; [see Methods]). First, miR-155 levels were measured by qPCR from all cells. The three cell lines with high NF-κB activity (due to LMP1 expression [IB4] or loss of IκBα [KMH2, and L428]) had substantially more miR-155 than the six cell lines with low NF-κB activity (Figure 4A).

Nuclear extracts were then prepared from these nine cell lines and analyzed for expression of p65, REL, and PARP (as a normalizing control) by Western blotting (Figure 4B). Two of the three cell lines with high NF-κB activity and high miR-155 expression, namely IB4 and KMH2, showed the highest levels of nuclear p65; the third high NF-κB/high miR155 cell line, L428, did not have high levels of nuclear p65. However, L428 cells, as well as IB4 and KMH2 cells, have high levels of nuclear REL. Among the six cell lines with low NF-κB/low miR155, all had low levels of nuclear p65 and five of six had low levels of nuclear REL; only SUDHL4 cells had high nuclear REL (Figure 4B). These results show that increased nuclear NF-κB protein (p65 or REL) generally correlates with high miR-155 expression.

## Discussion

This report demonstrates that *MIR155HG* proximal promoter region can serve as a direct target for activation by NF-κB. Reporter gene assays identified an NF-κB p50/p65-responsive site located ~178 nt upstream of the *MIR155HG* TSS. Moreover, *MIR155HG* pre-mRNA expression and p50/p65 binding to the -178 site can be induced by LPS treatment in BJAB cells. Lastly, miR-155 expression generally correlates with levels of nuclear NF-κB proteins (p65 or REL) in a panel of human B-lymphoma cell lines.

We report that LPS rapidly induces both *MIR155HG* pre-mRNA (Figure 1B) and mature miR-155 (Figure 1C) in BJAB cells. The use of one intron and one exon primer to measure *MIR155HG* pre-mRNA levels by qPCR suggests that LPS induces new transcription of *MIR155HG*, which then leads to increased levels of mature miR-155, and that, for example, induction of miR-155 levels is not due to a post-transcriptional process. This finding is consistent with our previous report [7] that LPS induces mature *MIR155HG* mRNA (using two exon primers) and miR-155 in BJAB cells. Taken together with our findings that LPS treatment of BJAB cells induces nuclear translocation of p65 (Figure 1D) and binding of p50/p65 to the -178 site in the *MIR155HG* promoter (Figure 1D), and that p50/p65 can activate the *MIR155HG* promoter in reporter assays (Figure 2), these results strongly suggest that LPS induces transcription of the *MIR155HG* promoter through binding of induced NF-κB p50/p65 to the -178 site, which ultimately results in increased levels of miR-155.

Although some qPCR guidelines prescribe the use of multiple reference genes for qPCR analyses [40], we have not done that. Nevertheless, we think it likely that LPS does induce *MIR155HG* transcription and miR-155 levels given that we have used different normalization transcripts and different RT-PCR approaches to measure the distinct *MIR155GH*-generated RNAs, and all methods yielded similar results. First, *GAPDH* was used as the reference transcript for measuring *MIR155HG* pre-mRNA levels (using intron-exon primers) by qPCR [Figure 1B]. Second, c-*rel* and *GAPDH* transcripts were not affected by LPS treatment in BJAB cells where induction of the mature *MIR155HG* transcript was measured by conventional RT-PCR using two exon primers [7]. Third, 5S RNA was used to normalize miR-155 levels using a small RNA qPCR method ([7,35]; Figure 1C). In addition, as measured by Northern blotting, it was previously shown that LPS does not change the levels of *GAPDH* mRNA [41]. Details of the methods we used to isolate and process mRNA and to perform qPCR experiments are included with Additional file 1: Table S1 and Additional file 4: Table S2.

Transcriptional regulation of miR-155 has been widely regarded to be an NF-κB-dependent process, although no compelling evidence showing a direct and transcriptionally functional interaction of NF-κB with the promoter/enhancer region of *MIR155HG* has been previously produced. Two studies [21,22] used reporter gene analyses to identify *cis* elements in the *MIR155HG* promoter that are required for transcriptional activation by the EBV LMP1 protein, which is an inducer of both NF-κB and AP-1 [42,43]. One study [21] claimed that a conserved AP-1 site 40 nt upstream of the *MIR155HG* TSS is required for high level miR-155 expression in EBV^+^ B-lymphoblast cell line JY. A second report [22] showed that the ability of the EBV LMP1 protein to enhance expression from the *MIR155HG* promoter in reporter assays in primary mouse embryo fibroblasts requires two NF-κB binding sites located more than 1100 nt upstream of the TSS. In contrast, we saw no effect of mutation of the -1150 site (analyzed in [22]) on p65-enhanced activation of the *MIR155HG* promoter in COS-1 cells. It is not clear whether the difference between those two studies and our study is due to the cell types used in the reporter assays or the method used to enhance NF-κB activity (i.e., LMP1-induced activation of NF-κB vs direct transfection of NF-κB family proteins).

Based on protein-binding microarrays using recombinant proteins [44], the -178 site (5’GGGAAAACAC3’) is not predicted to be a strong p65 binding site, although it is a reasonably good p50 binding site due primarily to the G-rich nature of the 5’ half-site. Consistent with those data, we have found that p50 homodimers readily bind the -178 site, whereas p65 homodimers do not (Figure 3B). Thus, in cells it is likely that p50/p50 homodimers strongly bind the -178 site to repress transcription in the absence of NF-κB p50/p65 activating signals. That p50/p50 homodimers can repress transcription from the *MIR155HG* promoter is consistent with our finding that high levels of transfected p50 alone reduce expression of the *MIR155HG* reporter in both wild-type and *nfkb1*^-/-^*nfkb2*^-/-^*relb*^-/-^*crel*^-/-^ 3T3 cells (Figure 3D). Once NF-κB is activated, p50/p65 heterodimers likely bind to the -178 site, a process that is facilitated by the high affinity of p50 for the 5’ half-site. Other promoters containing sites that selectivity bind p50/p65 heterodimers include those of the *pro-IL1β*, *ICAM-1*, and *CD166* genes [45-47].

Among aggressive B-cell lymphomas such as DLBCLs, there is a positive correlation between high NF-κB activity and high miR-155 expression [7,12-15]. Moreover, treatment of some B-lymphoma cell lines with NF-κB signaling inhibitors results in reduced miR-155 expression [48,49]. In our study, three DLBCL cell lines known to have high NF-κB activity also exhibited high expression of miR-155 (Figure 4A). IB4 and KMH2 cells have high nuclear p65 and REL, whereas L428 cells have high nuclear REL but low nuclear p65 (Figure 4B). Similar to L428 cells, RC-K8 cells have inactivating mutations in IκBα, high miR-155 expression, and exceptionally high levels of nuclear REL (but little or no nuclear p65) [33]. Moreover, over-expression of an oncogenically activated mutant REL in BJAB cells results in increased expression of *MIR155HG*[32]. SUDHL4 cells are unusual in that they have relatively high levels of nuclear REL protein (Figure 4B; [25,50]), but express low levels of miR-155 (Figure 4A) and have an overall low NF-κB gene expression profile [25]. Thus, nuclear REL may contribute to increased miR-155 expression in some (but not all) B-lymphoma cells. Nevertheless, REL did not activate the proximal *MIR155HG* promoter in COS-1 cell reporter gene assays (Figure 1A). Therefore, there may be REL-dependent sites outside of the 1494 bp fragment analyzed here that can regulate *MIR155HG* expression. Alternatively, REL may interact with factors in some B-lymphoma cells (which are not present in COS-1 or SUDHL4 cells) to enhance *MIR155HG* expression through its proximal promoter.

We used the proximal upstream (-1494 bp) region of the *MIR155HG* promoter to investigate the regulation of miR-155 expression by NF-κB. Although only approximately 20-30% of induced p65 binding sites are located within ~2 kbp of transcription start sites [51,52], approximately 50-60% of the coactivator p300/CBP binding is located within proximal promoters of actively transcribed genes [53,54], suggesting that much of the biologically relevant transcription-inducing p65 binding occurs in sequences proximal to the 5’ end of genes. Nevertheless, it has been reported that 28-40% of induced p65 binding sites are located in introns of known genes [51,52]. Indeed, the two introns of the human *MIR155HG* gene contain several predicted NF-κB sites (using the PROMO and TRED transcription factor binding site programs [36,37]). One predicted site in intron 1 and three in intron 2 have scores for NF-κB binding that are as high as the -178 site that we have described. Thus, it is possible that there are other p65 (or REL) sites that contribute to NF-κB-dependent regulation of *MIR155HG*/miR-155 expression. Moreover, in the mouse *MIR155HG* promoter there does not appear to be an NF-κB site that corresponds to the -178 site (that we have identified in the human *MIR155HG* promoter), suggesting that there may be differences in the regulation of miR-155 expression by NF-κB between the two species.

## Conclusions

The results presented herein show that NF-κB p50/p65 can directly activate the human *MIR155HG* promoter, consistent with the many studies that have reported a correlation between high or induced NF-κB activity and increased expression of miR-155 (reviewed in [2,9,19]). Due to the contributions of miR-155 to immune cell function and malignancy, understanding how *MIR155HG* expression is controlled could lead to new therapeutic approaches.

## Abbreviations

BIC: *B-cell integration cluster*; DLBCL: Diffuse large B-cell lymphoma; DMEM: Dulbecco’s modified Eagle’s medium; EMSA: Electrophoretic mobility shift assay; FBS: Fetal bovine serum; kbp: kilobase pairs; LPS: Lipopolysaccharide; miR: microRNA; nt: nucleotides; PCR: Polymerase chain reaction; PEI: Polyethylenimine; REL: Human c-Rel transcription factor; TSS: Transcription start site(s).

## Competing interests

The authors declare that they have no competing interests.

## Authors’ contributions

RCT planned and carried out the majority of the experiments. IV performed tissue culture experiments and reporter gene assays. RCT and TDG participated in the design of the study and wrote the manuscript. All authors read and approved the final manuscript.

## Supplementary Material

Additional file 1: Table S1 Primers used and their sequences. Click here for file

Additional file 2: Figure S1 Truncation mutants of the *MIR155HG* promoter were made via PCR, resulting in the creation of -530-MIR155HG and -91-MIR155HG luciferase reporter plasmids. Reporter assays were performed in COS-1 cells transfected with the pGL3-based -1494 bp *MIR155HG* promoter reported plasmid (WT-MIR155HG) or with indicated truncation mutant and with pcDNA vector control or pcDNA-FLAG-p65. Values were normalized to the pcDNA control (1.0). The * indicates p < 0.002. Click here for file

Additional file 3: Figure S2 Reporter assays were performed in COS-1 cells transfected with the pGL3-based wild-type *MIR155HG* promoter reported plasmid (WT-MIR155HG) or an AP-1 mutant (AP-1mut-MIR155HG) with either pcDNA alone or pcDNA-p65. Values for each transfection were normalized to RSV-renilla and then normalized to pcDNA alone (1.0). Click here for file

Additional file 4: Table S2 MIQE checklist for qPCR methods and analysis. Click here for file
