# Supplementary material for: Identification of an NF-κB p50/p65-responsive site in the human MIR155HG promoter
Source: BMC Mol Biol. 2013 Sep 23;14:24. doi: 10.1186/1471-2199-14-24 (PMC3849010; doi:10.1186/1471-2199-14-24)
Supplement: Additional file 2: Figure S1 — Truncation mutants of the MIR155HG promoter were made via PCR, resulting in the creation of -530-MIR155HG and -91-MIR155HG luciferase reporter plasmids. Reporter assays were performed in COS-1 cells transfected with the pGL3-based -1494 bp MIR155HG promoter reported plasmid (WT-MIR155HG) or with indicated truncation mutant and with pcDNA vector control or pcDNA-FLAG-p65. Values were normalized to the pcDNA control (1.0). The * indicates p < 0.002. [file 1471-2199-14-24-S2.pdf]

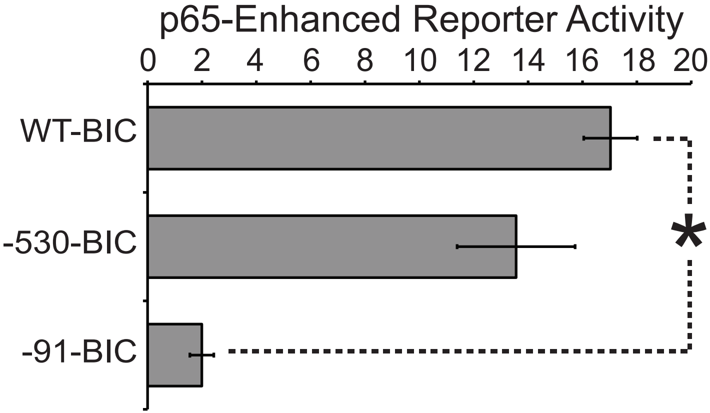

Supplemental Figure S1

Truncation mutants of the MIR155HG promoter were made via PCR, resulting in the creation of -530-MIR155HG and -91-MIR155HG luciferase reporter plasmids. Reporter assays were performed in COS-1 cells transfected with the pGL3-based -1494 bp MIR155HG promoter reported plasmid (WT-MIR155HG) or with indicated truncation mutant and with pcDNA vector control or pcDNA-FLAG-p65. Values were normalized to the pcDNA control (1.0). The * indicates $p<0.002$.
