# Supplementary material for: Identification of an NF-κB p50/p65-responsive site in the human MIR155HG promoter
Source: BMC Mol Biol. 2013 Sep 23;14:24. doi: 10.1186/1471-2199-14-24 (PMC3849010; doi:10.1186/1471-2199-14-24)
Supplement: Additional file 3: Figure S2 — Reporter assays were performed in COS-1 cells transfected with the pGL3-based wild-type MIR155HG promoter reported plasmid (WT-MIR155HG) or an AP-1 mutant (AP-1mut-MIR155HG) with either pcDNA alone or pcDNA-p65. Values for each transfection were normalized to RSV-renilla and then normalized to pcDNA alone (1.0). [file 1471-2199-14-24-S3.pdf]

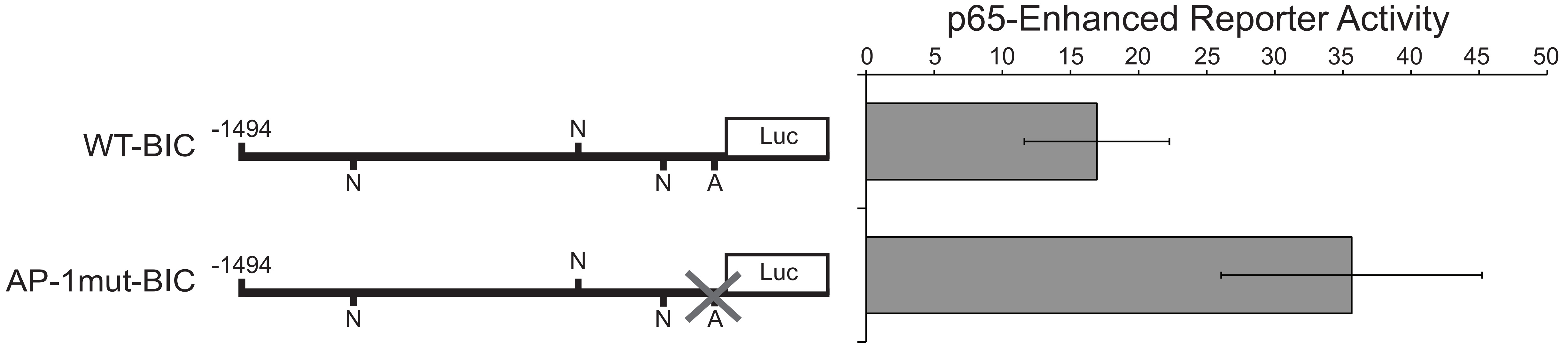

Supplemental Figure S2

Reporter assays were performed in COS-1 cells transfected with the pGL3-based wild-type MIR155HG promoter reported plasmid (WT-MIR155HG) or an AP-1 mutant (AP-1mut-MIR155HG) with either pcDNA alone or pcDNA-p65. The AP-1mut-MIR155HG plasimd was created by inserting an XhoI site in the middle of the AP-1 binding site, as previously performed (21). Values for each transfection were normalized to RSV-renilla and then normalized to pcDNA alone (1.0).
