# Supplementary material for: Identification of an NF-κB p50/p65-responsive site in the human MIR155HG promoter
Source: BMC Mol Biol. 2013 Sep 23;14:24. doi: 10.1186/1471-2199-14-24 (PMC3849010; doi:10.1186/1471-2199-14-24)
Supplement: Additional file 4: Table S2 — MIQE checklist for qPCR methods and analysis. [file 1471-2199-14-24-S4.pdf]

Table S2. MIQE checklist for qPCR methods and analysis

ITEM TO CHECK	IMPORTANCE	CHECKLIST
**EXPERIMENTAL DESIGN**		
Definition of experimental and control groups	**E**	Yes
Number within each group	**E**	3 samples/triplicate expts
Assay carried out by core lab or investigator's lab?	D	Investigator's lab
Acknowledgement of authors' contributions	D	Yes (separate section at end of text)
**SAMPLE**		
Description	**E**	Tissue culture cells
Volume/mass of sample processed	D	0.75 x 10^5 - 1 X 10^6 cells, depending on the cell line
Microdissection or macrodissection	**E**	Not applicable
Processing procedure	**E**	
If frozen - how and how quickly?	**E**	Yes, RNA frozen at -80 degrees immediately after isolation
If fixed - with what, how quickly?	**E**	Not applicable
Sample storage conditions and duration (especially for FFPE sample	**E**	Stored at -80 in RNAse-free water for 1-5 days
**NUCLEIC ACID EXTRACTION**		
Procedure and/or instrumentation	**E**	
Name of kit and details of any modifications	**E**	Not applicable
Source of additional reagents used	D	TRIzol reagent (Invitrogen), Chloroform
Details of DNase or RNAse treatment	**E**	RNA resuspended in DEPC-treated water and reverse transcriptase reactions were treated with RNAse; no DNase treatment
Contamination assessment (DNA or RNA)	**E**	Done by OD 260/280 determination; see 22, 23 below; also no reverse transcriptase control done for qPCR
Nucleic acid quantification	**E**	
Instrument and method	**E**	Nano-drop RNA extract
Purity (A260/A280)	D	Approximately 2.0 for each sample
Yield	D	0.5-4.5 ug/ul
RNA integrity method/instrument	**E**	Not performed
RIN/RQI or Cq of 3' and 5' transcripts	**E**	Not performed
Electrophoresis traces	D	Not performed
Inhibition testing (Cq dilutions, spike or other)	**E**	Not performed
**REVERSE TRANSCRIPTION**		
Complete reaction conditions	**E**	Supplied in M&M and Supplemental Table 1
Amount of RNA and reaction volume	**E**	See Supplemental Table 1: 2 µg in 30 µl total or 0.5 µg in 15 µl total, depending on the reaction
Priming oligonucleotide (if using GSP) and concentration	**E**	Not applicable
Reverse transcriptase and concentration	**E**	See Supplemental Table 1: 1 µl of Promega M-MLV-RT or 1 µl Applied Biosystems MultiScribe Reverse Transcriptase, depending on the reaction
Temperature and time	**E**	See Supplemental Table 1: 37°C for 1 h or 30 min at 16°C, 30 min at 42°C, and 5 min at 85°C, depending on the reaction.
Manufacturer of reagents and catalogue numbers	D	See Supplemental Table 1
Cqs with and without RT	D*	Not performed
Storage conditions of cDNA	D	Stored at -80 in RNAse-free water
**qPCR TARGET INFORMATION**		
If multiplex, efficiency and LOD of each assay.	**E**	Not applicable
Sequence accession number	**E**	Not applicable
Location of amplicon	D	See M&M
Amplicon length	**E**	150-250 nts
In silico specificity screen (BLAST, etc)	**E**	Yes, BLAST and UCSC Browser analysis performed
Pseudogenes, retropseudogenes or other homologs?	D	None
Sequence alignment	D	Done
Secondary structure analysis of amplicon	D	Not performed
Location of each primer by exon or intron (if applicable)	**E**	See Supplemental Table 1
What splice variants are targeted?	**E**	None
**qPCR OLIGONUCLEOTIDES**		
Primer sequences	**E**	See Supplemental Table 1
RTPrimerDB Identification Number	D	Not applicable
Probe sequences	D**	Not applicable
Location and identity of any modifications	**E**	None
Manufacturer of oligonucleotides	D	Invitrogen
Purification method	D	Desalted, by manufacturer (Invitrogen)
**qPCR PROTOCOL**		
Complete reaction conditions	**E**	See M&M and Supplemental Table 1
Reaction volume and amount of cDNA/DNA	**E**	2 µl of cDNA prep in 30 µl total volumne, then 8.5 µl is loaded into three wells of a 384-well plate
Primer, (probe), Mg++ and dNTP concentrations	**E**	10 pmoles of each primer into 30 µl total as above
Polymerase identity and concentration	**E**	See line 63
Buffer/kit identity and manufacturer	**E**	See line 63
Exact chemical constitution of the buffer	D	See line 63
Additives (SYBR Green I, DMSO, etc.)	**E**	15 µl of SYBR-green (4368577, ABI) in 30 µl total as above
Manufacturer of plates/tubes and catalog number	D	MicroAmp Optical 384-Well Reaction Plate with Barcode (4309849, ABI)
Complete thermocycling parameters	**E**	See M&M
Reaction setup (manual/robotic)	D	Manual
Manufacturer of qPCR instrument	**E**	Applied Biosystems
**qPCR VALIDATION**		
Evidence of optimisation (from gradients)	D	None
Specificity (gel, sequence, melt, or digest)	**E**	Agarose gel to see single band for GAPDH control; see also ref. 7
For SYBR Green I, Cq of the NTC	**E**	Not performed
Standard curves with slope and y-intercept	**E**	Not performed
PCR efficiency calculated from slope	**E**	Not performed
Confidence interval for PCR efficiency or standard error	D	Standard Error
r2 of standard curve	**E**	Not performed
Linear dynamic range	**E**	Yes
Cq variation at lower limit	**E**	Not performed
Confidence intervals throughout range	D	Not performed
Evidence for limit of detection	**E**	Not performed
If multiplex, efficiency and LOD of each assay.	**E**	Not applicable
**DATA ANALYSIS**		
qPCR analysis program (source, version)	**E**	Microsoft Excel version 12.3.4
Cq method determination	**E**	Determined and analyzed by sequence detection software (SDS 2.2, ABI)
Outlier identification and disposition	**E**	Not performed
Results of NTCs	**E**	Not performed
Justification of number and choice of reference genes	**E**	See M&M and Discussion
Description of normalisation method	**E**	See M&M and Supplemental Table 1
Number and concordance of biological replicates	D	3
Number and stage (RT or qPCR) of technical replicates	**E**	3
Repeatability (intra-assay variation)	E	< 1.5%
Reproducibility (inter-assay variation, %CV)	D	Presented in Figures as Standard Error
Power analysis	D	Not performed
Statistical methods for result significance	**E**	ANOVA
Software (source, version)	E	AnalystSoft Inc. StatPlus version 5
Cq or raw data submission using RDML	**D**	Not done

Table 1. MIQE checklist for authors, reviewers and editors. All essential information (E) must be submitted with the manuscript. Desirable information (D) should be submitted if available. If using primers obtained from RTPrimerDB, information on qPCR target, oligonucleotides, protocols and validation is available from that source.

*: Assessing the absence of DNA using a no RT assay is essential when first extracting RNA. Once the sample has been validated as RDNA-free, inclusion of a no-RT control is desirable, but no longer essential.

**: Disclosure of the probe sequence is highly desirable and strongly encouraged. However, since not all commercial pre-designed assay vendors provide this information, it cannot be an essential requirement. Use of such assays is advised against.
